# Expansion of a SARS-CoV-2 Delta variant with an 872 nt deletion encompassing *ORF7a*, *ORF7b* and *ORF8*, Poland, July to August 2021

**DOI:** 10.2807/1560-7917.ES.2021.26.39.2100902

**Published:** 2021-09-30

**Authors:** Natalia Mazur-Panasiuk, Lukasz Rabalski, Tomasz Gromowski, Grzegorz Nowicki, Michal Kowalski, Witold Wydmanski, Piotr Szulc, Maciej Kosinski, Karolina Gackowska, Natalia Drweska-Matelska, Jakub Grabowski, Anna Piotrowska-Mietelska, Boguslaw Szewczyk, Krystyna Bienkowska-Szewczyk, Jakub Swadzba, Pawel Labaj, Maciej Grzybek, Krzysztof Pyrc

**Affiliations:** 1 Virogenetics Laboratory of Virology, Malopolska Centre of Biotechnology, Jagiellonian University, Krakow, Poland; 2 Laboratory of Recombinant Vaccines, Intercollegiate Faculty of Biotechnology of University of Gdansk and Medical University of Gdansk, Gdansk, Poland; 3 Human Genome Research Variation Group, Malopolska Centre of Biotechnology, Jagiellonian University, Krakow, Poland; 4 genXone SA, Research & Development Laboratory, Suchy Las, Poland; 5 Bioinformatics Research Group, Malopolska Centre of Biotechnology, Jagiellonian University, Krakow, Poland; 6 Diagnostyka Medical Laboratories, Krakow, Poland; 7 Laboratory of Virus Molecular Biology, Intercollegiate Faculty of Biotechnology of University of Gdansk and Medical University of Gdansk, Gdansk, Poland; 8 Department of Tropical Parasitology, Institute of Maritime and Tropical Medicine, Medical University of Gdansk, Gdynia, Poland

**Keywords:** SARS-CoV-2, variant, deletion, pandemic, detection

## Abstract

Routine genomic surveillance on samples from COVID-19 patients collected in Poland during summer 2021 revealed the emergence of a SARS-CoV-2 Delta variant with a large 872 nt deletion. This change, confirmed by Sanger and deep sequencing, causes complete loss of *ORF7a*, *ORF7b*, and *ORF8* genes. The index case carrying the deletion is unknown. The standard pipeline for sequencing may mask this deletion with a long stretch of N’s. Effects of this deletion on phenotype or immune evasion needs further study.

Between 1 July and 16 August 2021, 316 severe acute respiratory syndrome coronavirus 2 (SARS-CoV-2) genomes from 13 of 16 Polish voivodships from isolates obtained during routine surveillance activities, were deposited by our group in the GISAID database [1]. Of these, 31 sequences, all of which belonged to the SARS-CoV-2 variant of concern (VOC) Delta (Phylogenetic Assignment of Named Global Outbreak (Pango) lineage designation B.1.617.2) AY.4 sublineage designation, showed an unusual pattern i.e. a long stretch of N’s, indicating a weak or undetermined nucleotide signal, in the region corresponding to the open reading frame *(ORF)7a*, *ORF7b* and *ORF8* genes in the reference SARS-CoV-2 genome (GenBank NC_045512). 

Most of these unique sequences (27/31) originated from coronavirus disease (COVID-19) patient samples collected in the Malopolska voivodship in southern Poland, between 17 July and 11 August 2021. Our aim was to investigate the emergence of the unusual sequence pattern in SARS-CoV-2 sequences from Poland. 

## Clinical presentation

Clinical data were available for 24 of the 31 patients with atypical Delta VOC sequences. COVID-19 was suspected in all patients, since they showed clinical symptoms compatible with the disease; all were confirmed by a SARS-CoV-2 RT-qPCR diagnostic test, referred by a general practitioner. The median age was 31 years (range: 4 months–84 years), with an equal number of females (n = 12) and males (n = 12). Symptoms included fever (n = 23), cough (n = 15), headache (n = 10), weakness (n = 10), muscle pain (n = 10), runny nose (n = 7), loss of smell (n = 3), loss of taste (n = 2), sore throat (n = 1), diarrhoea (n = 1), dyspnoea (n = 1) and breathing difficulties (n = 1). Seven patients were hospitalised, and four needed oxygen supplementation. Fifteen of the 24 patients were not vaccinated or partially vaccinated (one dose, various mRNA and non-mRNA vaccines) against SARS-CoV-2; of the seven hospitalised patients, five were not vaccinated. None of the patients travelled abroad before the COVID-19 symptoms developed.

## PCR and sequencing analysis

For sample collection, nasopharyngeal swab samples were taken according to the WHO guidelines and in line with the protocols approved by the Polish Ministry of Health [2]. RNA was extracted by the various laboratories, accordingly to their validated, routinely performed methods used for SARS-CoV-2 diagnostic. Extracted RNA was selected randomly, was frozen at −80°C and sent from collection to sequencing laboratories within 2 weeks. The sequencing was performed with Oxford Nanopore Technologies (ONT) platform (Oxford, United Kingdom) using the ARTIC amplicon v3 and Midnight whole genome sequencing protocols [3-5]. Sequencing results showed a long stretch of N’s within the region covering the *ORF7a, ORF7b* and *ORF8* genes. Since the technique is based on the targeted amplification, this observed artefact i.e. a long stretch of N’s, was suspected to be the result of a short insertion–deletion mutation (indel) or single nucleotide polymorphism (SNP) within the primer target sites. We selected 14 specimens for further genetic analyses. To exclude an unspecific primer binding event, we tested the primer pair (e.g. 90_Left and 93_Right primers, which was used for the ARTICv3 protocol and overlapped the region of interest) for SARS-CoV-2 genome amplification (Table 1).

**Table 1:** Primer pairs used for amplification and Sanger sequencing of selected SARS-CoV-2 specimens, Poland, 17 July–11 August 2021

Pair	Sequencing protocol	Primer name	Primer sequence (5’ to 3’)
Pair 1	ARTICv3	90_Left	ACACAGACCATTCCAGTAGCAGT
		93_Right	AGGTCTTCCTTGCCATGTTGAG
Pair 2	Midnight	27_Left	TGGATCACCGGTGGAATTGCTA
		28_Right	GTTTGGCCTTGTTGTTGTTGGC

SARS-CoV-2: severe acute respiratory syndrome coronavirus 2.

ARTICv3 [4,5] and Midnight protocols [3] are whole genome sequencing procedures based on amplification of specific SARS-CoV-2 genome regions with the use of sets of primers.

**Figure 1:**
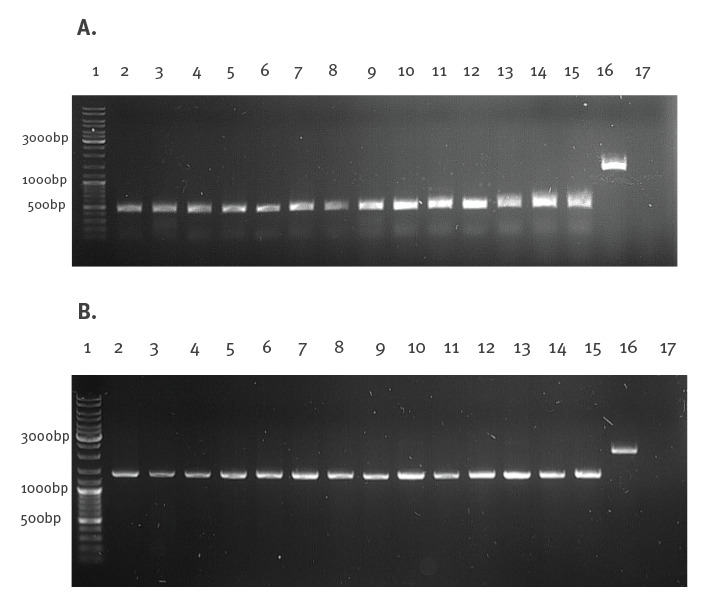
Amplified PCR fragments from isolates expressing a unique SARS-CoV-2 Delta variant sequence, Poland, 17 July–11 August 2021 (n = 14)

Agarose gel electrophoresis showed that the amplification products obtained from the ARTICv3 protocol primers [4,5] were ca 500 bp, rather than the expected size of 1,328 bp (Figure 1).

SARS-CoV-2: severe acute respiratory syndrome coronavirus 2.

PCR was performed with (A) ARTICv3 primers (90_Left, 93_Right) [4,5] and (B) Midnight primers (27_Left, 28_Right) [3] and analysed with agarose gel electrophoresis. Isolates with unique SARS-CoV-2 Delta variant sequences (lanes 2–15) reveal a band at ca 500 bp (A) and at ca 1400 bp (B) rather than the expected size of 1,328 bp (lane 16, A) or 2,265 bp (lane 16, B). Lane 1: GeneRuler DNA ladder mix (Thermo Fisher Scientific); Lane 2: sample hCoV-19/Poland/PL_P3804/2021; Lane 3: sample hCoV-19/Poland/PL_P3711/2021; Lane 4: sample hCoV-19/Poland/PL_P3699/2021; Lane 5: sample hCoV-19/Poland/PL_P3705/2021; Lane 6: sample hCoV-19/Poland/PL_P3687/2021; Lane 7: sample hCoV-19/Poland/PL_P3686/2021; Lane 8: sample hCoV-19/Poland/PL_P3695/2021; Lane 9: sample hCoV-19/Poland/PL_P3696/2021; Lane 10: sample hCoV-19/Poland/PL_P3688/2021; Lane 11: sample hCoV-19/Poland/PL_P3693/2021; Lane 12: sample hCoV-19/Poland/PL_P3704/2021; Lane 13: sample hCoV-19/Poland/PL_P3794/2021; Lane 14: sample hCoV-19/Poland/PL_P3710/2021; Lane 15: sample hCoV-19/Poland/PL_P3709/2021; Lane 16: SARS-CoV-2 Delta AY.4 control sample, hCoV-19/Poland/PL_P3707/2021; Lane 17: negative control.

This unexpected result suggested a large ca 850 nt deletion in these isolates. In order to exclude mispriming events, the second pair of primers originating from the Midnight protocol [3] (e.g. 27_Left, 28_Right) (Table 1)(Figure 1) was used to confirm the deletion. Similar to the ARTICv3 primer pair, the products of the second primer set were ca 850 nt shorter than the expected 2,265 bp. 

**Figure 2:**
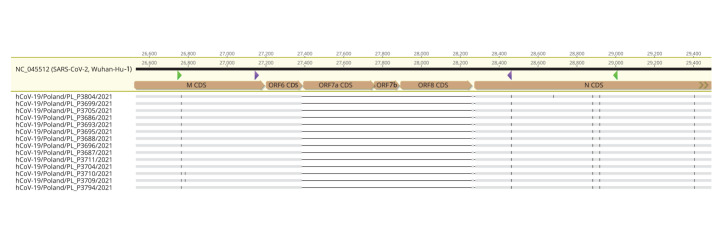
Alignment of the consensus sequences of isolates lacking the 872 nt region to the reference SARS-CoV-2 genomic sequence, Poland, 17 July–11 August 2021 (n = 14)

CDS: coding sequence; ORF: open reading frame; SARS-CoV-2: severe acute respiratory syndrome coronavirus 2.

The obtained amplicons were subjected to in-house ONT sequencing using native barcoding for the whole amplicon. In parallel, the products obtained in the first round of RT-PCR were subjected to Sanger sequencing. Both methods confirmed a deletion of 872 nt, which stretches between nucleotide positions 27,385 and 28,256 of the reference genome. This sizable alteration causes a complete loss of *ORF7a*, *ORF7b*, and *ORF8* genes (Figure 2). The obtained sequences were deposited in the GISAID database under the following accession numbers: hCoV-19/Poland/PL_P3686/2021; hCoV-19/Poland/PL_P3688/2021; hCoV-19/Poland/PL_P3693/2021; hCoV-19/Poland/PL_P3695/2021; hCoV-19/Poland/PL_P3696/2021; hCoV-19/Poland/PL_P3699/2021; hCoV-19/Poland/PL_P3704/2021; hCoV-19/Poland/PL_P3705/2021; hCoV-19/Poland/PL_P3709/2021; hCoV-19/Poland/PL_P3711/2021; hCoV-19/Poland/PL_P3794/2021; hCoV-19/Poland/PL_P3804/2021; hCoV-19/Poland/PL_P3687/2021; hCoV-19/Poland/PL_P3710/2021.

The consensus sequences were combined based on Sanger and Oxford Nanopore Technology sequencing. The triangles marked within the reference sequence (yellow-shaded in the top) represent the primer binding sites of the two primer pairs: purple arrows (90_L and 93_R; ARTICv3 protocol [4,5]); green arrows (27_L and 28_R; Midnight protocol [3]).

## Rapid barcoding error exclusion

Such methods of high-throughput whole genome sequencing like ONT of SARS-CoV-2 are usually performed in a 96-well plate format using the barcoding approach, which is time- and cost-effective, but may lead to sporadic misclassification of some reads between the samples. This mistake could be wrongly reported as ‘quasi-species’. To exclude artefacts related to the barcoding procedure and to detect SARS-CoV-2 quasi-species, selected samples were run individually on a single flow cell. Moreover, during our analyses, we used a highly accurate ONT Guppy 5.0.11 super high accuracy (SUP) base-calling algorithm supported by detecting a barcode from both ends. None of the reads were mapped to the deleted region, which supported the hypothesis that isolates containing the 872 nt deletion are not mixed with the wild-type virus, thus representing a pure population of these new Delta AY.4 isolates with the 872 nt deletion.

## Viral load

**Figure 3:**
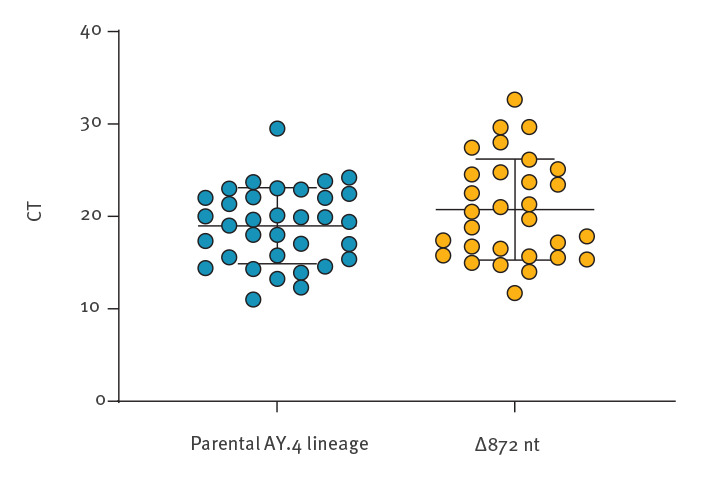
Comparison of viral load between the SARS-CoV-2 Delta AY.4 lineage (n = 34) and the Delta AY.4 isolates with the 872 nt deletion (n = 30), Poland, 17 July–11 August 2021

CT: cycle threshold; SARS-CoV-2: severe acute respiratory syndrome coronavirus 2.

The viral load, expressed as a mean cycle threshold (CT) value obtained during diagnostic RT-qPCR, was available for 30 samples for three different commonly used gene targets (*E*: envelope gene, *N*: nucleoprotein gene, *ORF1a*: *ORF1a* gene). To assess the potential influence of the 872 nt deletion on the virus transmissibility, the CT was compared with selected other Delta variant AY.4 lineage samples (n = 34) confirmed in Poland between 15 July and 16 August 2021 (Figure 3). The viral loads did not vary between the groups, and thus it may be assumed that these Delta AY.4 isolates with the 872 nt deletion show similar transmissibility to the parental AY.4 lineage.

Each point refers to single sample, for which the CT value was calculated as a mean CT obtained from three target genes (*E, N, ORF1a*) obtained during routine diagnostic RT-qPCR. CT values are compared between SARS-CoV-2 AY.4 samples (n = 34) and Delta AY.4 isolates with the 872 nt deletion (n = 30). Error bars indicate standard deviation of the mean. The difference is statistically insignificant (p > 0.05, unpaired t-test).

## Ethical statement

No ethical approval was required for this study as samples were collected for routine surveillance.

## Discussion and conclusions

SARS-CoV-2 VOCs have appeared in Poland in a pattern similar to that observed in other European countries. From July 2021, the Delta VOC has been the dominant circulating variant. Similar to other RNA viruses, the SARS-CoV-2 genome evolves quite rapidly, indicated by a 1.1 × 10^3^ substitution/site/year mutation rate [6]. Numerous changes within SARS-CoV-2 genomes are continuously reported, from minor SNPs to extensive deletions, which have also been reported for other coronaviruses e.g. Middle East respiratory syndrome coronavirus (MERS-CoV) [7]. Regardless of size, all variations may impair the protein structure and function, which could cause changes in the virus phenotype, infectivity, infectiousness, illness severity, or host immune response. The detected 872 nt deletion leads to complete loss of three accessory proteins encoded by the *ORF7a*, *ORF7b*, and *ORF8* genes.

The gene encoding accessory protein ORF8 shows high variability, and multiple alterations including SNPs, short indels causing frameshifts and partial or complete gene deletions have been reported to date [8-10]. In recombinant SARS-CoV-1, the truncation of ORF8 led to gradual virus attenuation in vitro [7]. In SARS-CoV-2, the accessory protein encoded by *ORF8* has a function related to evasion of the host adaptive immune response via downregulation of MHC-1 (major histocompatibility complex) [11]. Moreover, ORF8 modulates the host’s interferon-mediated antiviral response [12]. However, the importance of ORF8 in vivo remains unresolved. While recombinant ΔORF8 SARS-CoV-2 viruses produced smaller plaques than the wild type (WT) in vitro, the viral growth kinetics remained unchanged [10,13]. Furthermore, human angiotensin converting enzyme 2 (hACE2)-transgenic mice infected with ΔORF8 recombinants showed similar pathological lesions and mortality to the WT strain, suggesting that ORF8 does not contribute to virus pathogenicity [13] An observational human cohort study revealed that individuals infected with SARS-CoV-2 lacking a functional *ORF8* gene was associated with lower odds of developing hypoxia requiring supplemental oxygen [14].

The deletions within the *ORF7a* and *ORF7b* region have also previously been reported [15-18]. ORF7a activates the nuclear factor kappa-light-chain-enhancer of activated B cells (NF-kappa B) pathway and induces proinflammatory cytokine expression [19,20]. The function of ORF7b is largely unknown, but a recent study has shown that the protein activates the type-I interferon (IFN) signalling pathway and promotes expression of IFN-beta, interleukin (IL)-6, and tumor necrosis factor (TNF)-alpha, which induce apoptosis [21]. Viruses lacking these genes also produced smaller plaques than WT viruses in vitro, but their growth kinetics were similar to the parental strain [13]. Based on studies in animal models, the proteins have a minor impact on pathological lesions and disease outcomes [13]. 

This is the first study, to our knowledge, confirming the emergence of such a large deletion within the SARS-CoV-2 genome, causing the complete loss of three consecutive ORF sequences, e.g. *ORF7a*, *ORF7b*, and *ORF8*. The clinical data obtained from 25 infected individuals suggest a typical course of COVID-19, with mild to moderate symptoms. The origin of these Delta AY.4 isolates with the 872 nt deletion remains unknown, but their detection in a short period of time with high frequency in the Malopolska voivodship suggests that they may be maintained in the population. Based on GISAID data, during recent weeks (September 2021), more than 70 similar sequences containing a stretch of N’s within the region corresponding to *ORF7a, ORF 7b* and *ORF 8* were confirmed in other areas in Poland, including Mazowieckie, Pomorskie and Swietokrzyskie voivodships. These sequences, similar to those presented here, could also contain the 872 nt deletion and thus, detection could be missed because of the bioinformatic algorithm leading to a long stretch of N’s at the position of the deleted genes. However, a re-analysis of raw sequencing data or Sanger sequencing is needed to confirm the deletion in these samples. Considering the potential spread of a virus strain similar to these Delta AY.4 isolates with the 872 nt deletion to the other regions in Poland, it may be hypothesised that its transmissibility has not been compromised. Unfortunately, the original swab samples were unavailable and the virus isolation was impossible at this point, but will be a subject for the future studies. The monitoring of this 872 nt deletion in clinical samples and the publicly available databases should be continued.
